# Lipids monitoring in *Scenedesmus obliquus* based on terahertz technology

**DOI:** 10.1186/s13068-020-01801-0

**Published:** 2020-09-16

**Authors:** Yongni Shao, Weimin Gu, Y ating Qiu, Shengfeng Wang, Yan Peng, YiMing Zhu, Songlin Zhuang

**Affiliations:** 1 Terahertz Technology Innovation Research Institute, Terahertz Spectrum and Imaging Technology Cooperative Innovation Center, Shanghai Key Lab of Modern Optical System, University of Shanghai for Science and Technology, Shanghai, 200093 China; 2 Shanghai Institute of Intelligent Science and Technology, Tongji University, Shanghai, 200092 China

**Keywords:** Terahertz, Raman spectroscopy, Microalgae, Lipids

## Abstract

**Background:**

Microalgae are considered as a source of low pollution and renewable fuel due to their ability to synthesize an abundance of lipids. Conventional methods for lipid quantification are time-consuming and chemically contaminated, while spectroscopic method combined with mathematical model is much more attractive due to its ability of qualitative and quantitative analysis of material composition, in this sense, terahertz technology provides not only timely and non-destructive testing without chemical pollution, but also provides information on the functional group vibration mode and structure of the measured components. Therefore, terahertz technology is utilized in our investigation and proposed for microalgae metabolism detection.

**Results:**

The aim of this study was to use terahertz spectroscopy to observe lipid content in *Scenedesmus obliquus* (*S. obliquus*). We collected the THz spectra of *S. obliquus* which were cultivated under nitrogen stress and terahertz spectroscopy was used to analyze changes in substance components (lipids, proteins, carbohydrates and β-carotene). The PLS algorithm was used to model the terahertz data to distinguish the different lipid content of *S. obliquus* under nitrogen stress. The correlation coefficient of the prediction results of the lipid characteristic band modeling was above 0.991, and the root mean square error was less than 0.132. It indicated that terahertz technology can be used to discriminate *S. obliquus* cells under different nitrogen stress effectively. The correlation between the terahertz characteristic peak (9.3 THz) and the total lipid content determined by gravimetry reaches 0.960. The final results were compared with the commonly used spectroscopic methods for lipid observation (Raman spectroscopy).

**Conclusions:**

In this article, we demonstrated the effectiveness of terahertz spectroscopy to monitor changes in microalgae lipid content under nitrogen stress. Terahertz spectroscopy is more suitable for industrial production or ordinary laboratories which require intermediate result with low-frequency screening. When quantifying microalgae lipids, the constraint of terahertz spectroscopy is far less than that of Raman spectroscopy, and it is easier for operator to accurately quantify microalgae lipid. In addition, it is still in early stage for the study of microalgae using terahertz spectroscopy technology, there is still much potential for us to explore.

## Background

Nowadays, the global energy crisis continuously gets more and more critical, which has raised interest in looking for low pollution and renewable energy resources. Among the many new energy sources, biodiesel has become the world's fastest growing and the most widely used clean renewable energy. As an important renewable resource, algae has the advantages of high photosynthetic efficiency, strong environmental adaptability and short growth cycle [1]. In order to provide better strategies for process conditions, nutritional schemes, and metabolic engineering techniques, researchers have studied the microalgal metabolic processes [2]. For example, under nitrogen stress, changes in lipid accumulation in microalgae cells provide conditions for studying the mechanism of lipid accumulation [3–5].

When the biodiesel is produced by microalgae, the content of lipids and types of fatty acids in cells are the important considerations [6]. In this process, the screening of high-quality microalgae and the monitoring of culture process are important for the efficient conversion of lipids. If the lipid content and distribution under nitrogen stress can be observed, a comprehensive understanding of lipid accumulation in microalgae cells can be obtained. However, the conventional methods used for the quantitative analysis of microalgae lipids, e.g., gas chromatography–mass spectrometry (GC–MS) can accurately analyze lipid components, but the detection is time-consuming, and the sample preparation process is complicated, in the meantime it cannot analyze the lipid metabolism process in cells. The other technology, Nile red (NR) or BODIPY 505/515 for instance can show lipid distribution in cells, but the fluorescence intensity is affected by many factors, such as emission wavelength, dye concentration, cell density and staining time [7–9]. Therefore, it is necessary to find a fast and non-destructive method for detecting lipid content.

Spectroscopic method combined with mathematical model can be used for qualitative and quantitative analysis of material composition [10, 11], the technique provides not only timely and non-destructive testing without chemical pollution (such as extraction and esterification), but also information on the functional group vibration mode and structure of the measured components. For example, non-polar groups such as C=C, C–C, etc., have strong Raman activity [12]. Raman spectroscopy can reflect the differences in sample chemical composition and molecular structure at the molecular level, and achieve "fingerprint identification" of certain chemical bonds and functional groups in the molecule. The disadvantage of Raman spectroscopy is the strong Raman signal of pigments (β-carotene, Chlorophyll a, etc.), which overlaps with the Raman intensity of the lipid and interferes with measurement accuracy [13]. With the development of spectroscopy techniques, other spectroscopy techniques have also been explored to study microbial metabolism, such as terahertz (THz) spectroscopy.

THz frequency range is from 0.1 to 20 THz, which is between the microwave and IR regions. THz has the advantages of fast analyzing speed, no chemical pollution, and no complex sample processing. It is suitable for large-scale sample testing [14]. The molecular structure of a compound is identified based on its characteristic absorption, so that the qualitative and quantitative analysis of the substance can be performed [15, 16]. In contrast to the conventional IR and Raman spectra, which mainly reflect intramolecular vibrations, the spectral information in the terahertz region is rich in collective modes such as intermolecular and backbone vibrations, which is more directly related to the molecular structure [17–19]. Therefore, THz signal can be a useful complement to the Raman and IR spectroscopy for monitoring microbial metabolism. In the past, researchers have used THz to study microorganisms [20, 21], such as distinguishing *Escherichia coli*, *Bacillus subtilis* and *Bacillus* species. THz spectroscopy may become a new technique for detecting metabolites of microalgae cells, which is suitable for macroscopic detection in metabolic engineering [22–24]. However, due to the complexity of vegetative cell components, there are no obvious characteristic peaks in the cell spectrum [20], and related reports lack the quantification of metabolites in cells [14]. Researchers prefer to conduct separate studies of cellular components outside the cell, such as fatty acids and their analogues distinction [25, 26], protein-structure recognition [15], starch quantification [16], forgetting the research of terahertz in the process of microalgae metabolism. Therefore, it is of great attraction to use terahertz to study the metabolic process of microalgae lipids accumulation.

In this study, THz spectroscopy was used to investigate the accumulation of lipids in *Scenedesmus obliquus* (*S. obliquus*). Gravimetric method, NR and GC–MS were used to validate the change of lipids under nitrogen stress. We collected the THz spectra of *S. obliquus* which were cultivated under nitrogen stress at different time from day 0 to day 10 with 2 days’ collection interval, and identified the main components in the *S. obliquus* according to their THz characteristic peaks, including lipids, carbohydrates, carotenoids and proteins. Their variation rules of the content as a function of the growth days of *S. obliquus* were also analyzed. Principal component analysis (PCA) and partial least square (PLS) were used to distinguish and predict microalgae from different stress days. In addition, the correlation model between the total lipid content measured by THz spectroscopy and gravimetric method was built. Then the laser confocal micro-Raman spectrometer was used to visualize the *S. obliquus* at 0–10 days, respectively, and the correlation model between the total lipid content measured by Raman spectroscopy and gravimetric method was also built. Then the THz model was compared with the Raman model.

## Results

### Content and composition of lipids in *Scenedesmus obliquus*

**Fig. 1:**
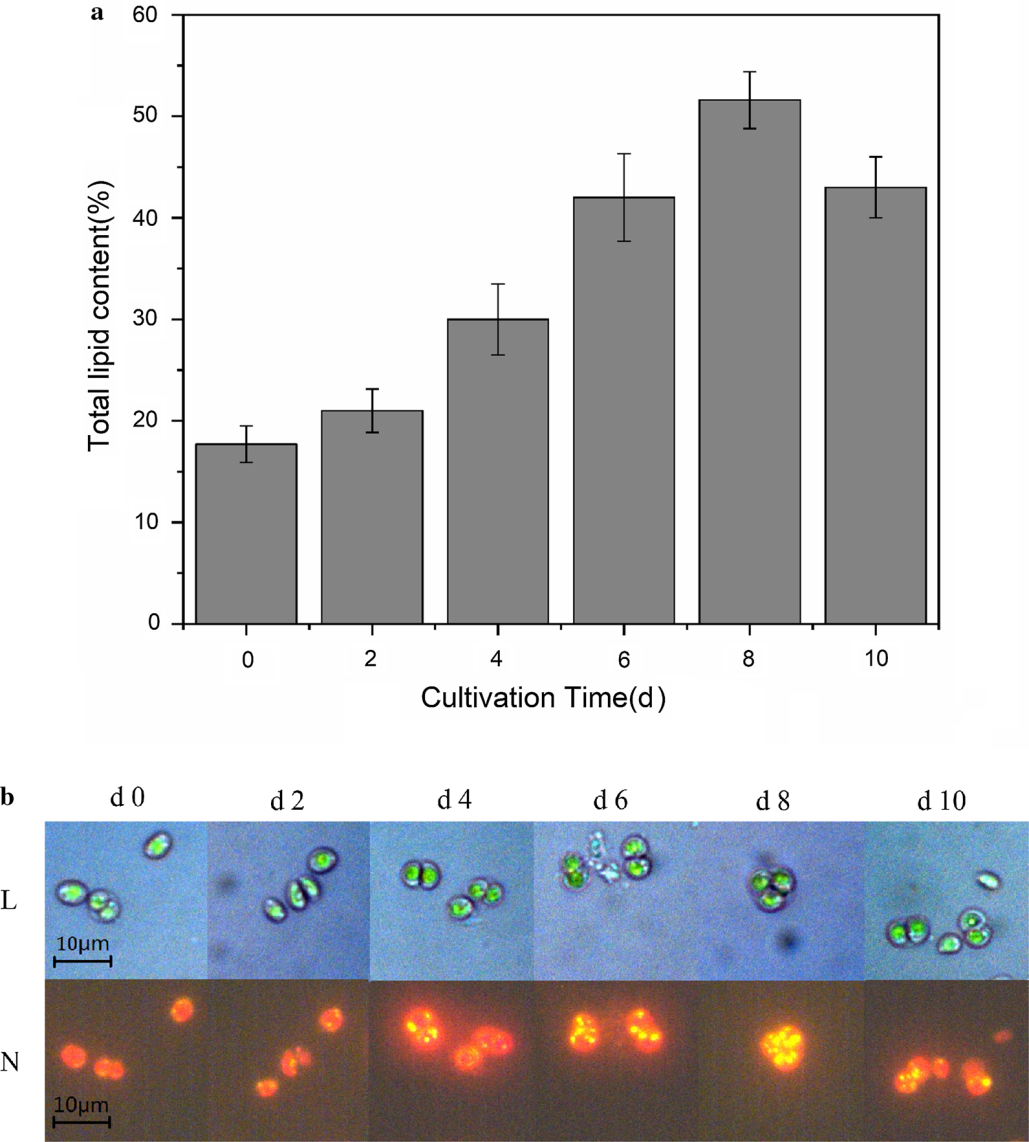
**a** Gravimetric methods for lipid content determination and **b** NR fluorescent images of *S. obliquus* at different days

As shown in Fig. 1a, the variation rule of lipids was found out that it increases first and then followed by a reduction, it reaches the highest value on the 8th day. The total lipid content of *S. obliquus* was 17.7% on day 0. On the 8th day, the lipid content reached 51.6%, which was 2.9 times of the initial value. We also used GC–MS to analyze the proportion of fatty acids in lipids and the variation in the fatty acid profile of *S. obliquus* in stress culture are summarized (Table 1). The proportion of SFA has reduced by 45% and the proportion of UFA has increased by 12% from day 0 to 8. GC–MS combined with dry weight method showed that the dry weight ratio of UFA increased from 11.16 to 44.51%, while the dry weight ratio of SFA decreased from 6.90 to 6.28% from day 0 to day 8.

**Table 1:** Fatty acid composition of *S. obliquus*

Fatty acid	Fatty acid content (%)		
	Day 0	Day 4	Day 8
C14:0	8.22	3.24	1.77
C16:0	30.12	19.41	12.35
C16:3	1.80	2.58	2.67
C16:4	10.76	17.55	29.8
C18:1	9.25	12.14	6.41
C18:2	11.37	10.73	7.64
C18:3	24.50	27.10	29.77
C18:4	3.65	6.33	8.55
C20:5	0.33	0.92	1.34
SFA	38.34	22.65	12.38
UFA	61.66	77.35	87.62

### Microscopic examination of *Scenedesmus obliquus*

**Fig. 2:**
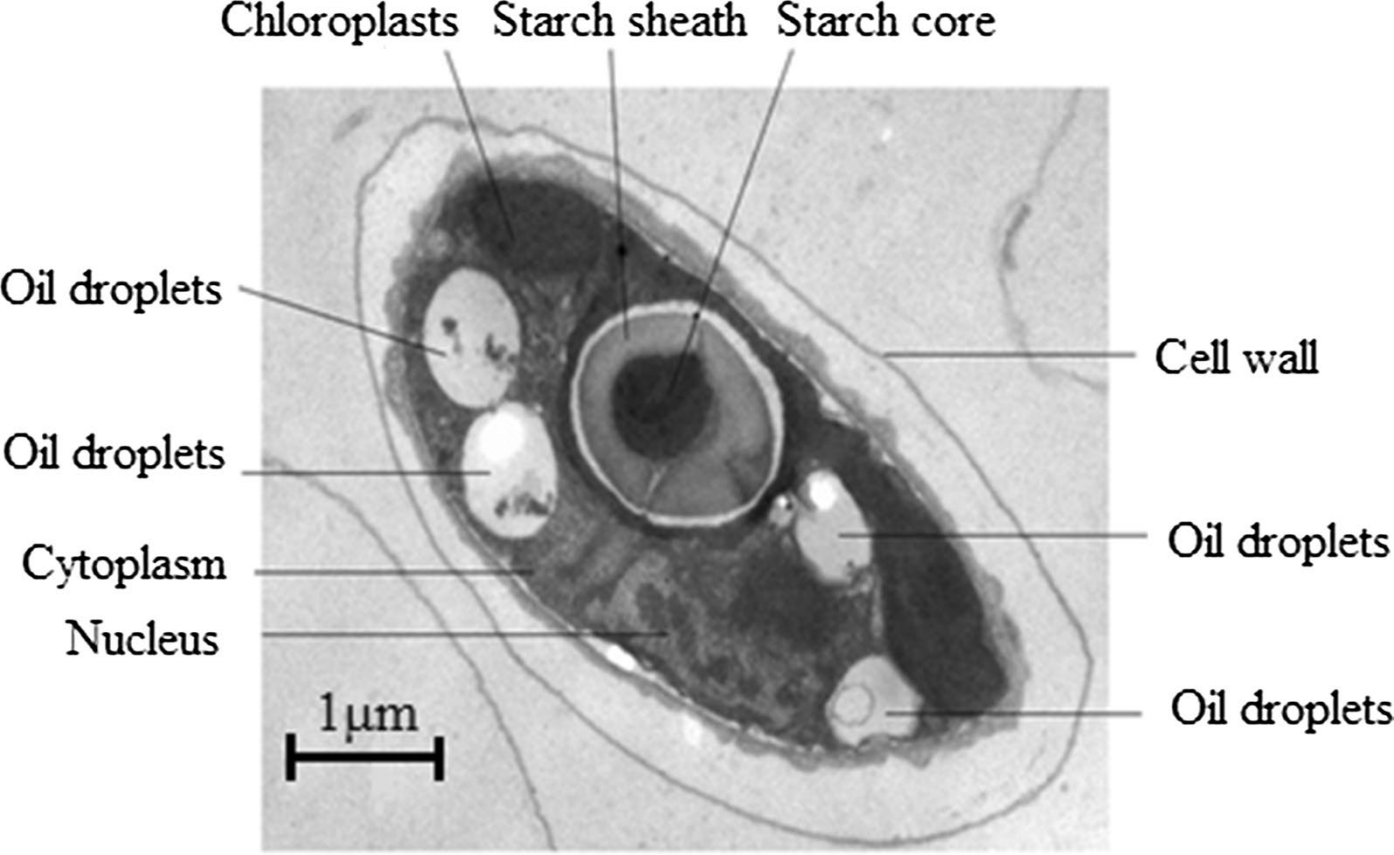
Transmission electron microscopic observation of *S. obliquus*

The image contains a lot of biological information. Obtain intuitive and clear images in the field of microbiological research, which can better analyze the characteristics and status of specific areas of cells or organisms, as well as the distribution of specific molecules. NR result indicated that lipid substances widely exist in cells and cells of *S. obliquus* appear yellow-orange in a fluorescence microscope after lipid staining. With the presence of accumulated oil droplets, they turn bright yellow. Figure 1b shows the fluorescence microscopic observation of different lipid accumulation in *S. obliquus* cells. However, due to the limited resolution of fluorescence microscopy, we used high-resolution TEM to supplement the observation of the microstructure of *S. obliquus*. The observed results are presented in Fig. 2. The study indicated that oil droplets were located near the cell membrane in the cytoplasm, and were mostly light gray circles or ellipses in TEM observation field.

### Observation of *Scenedesmus obliquus* lipids by Raman spectroscopy

**Fig. 3:**
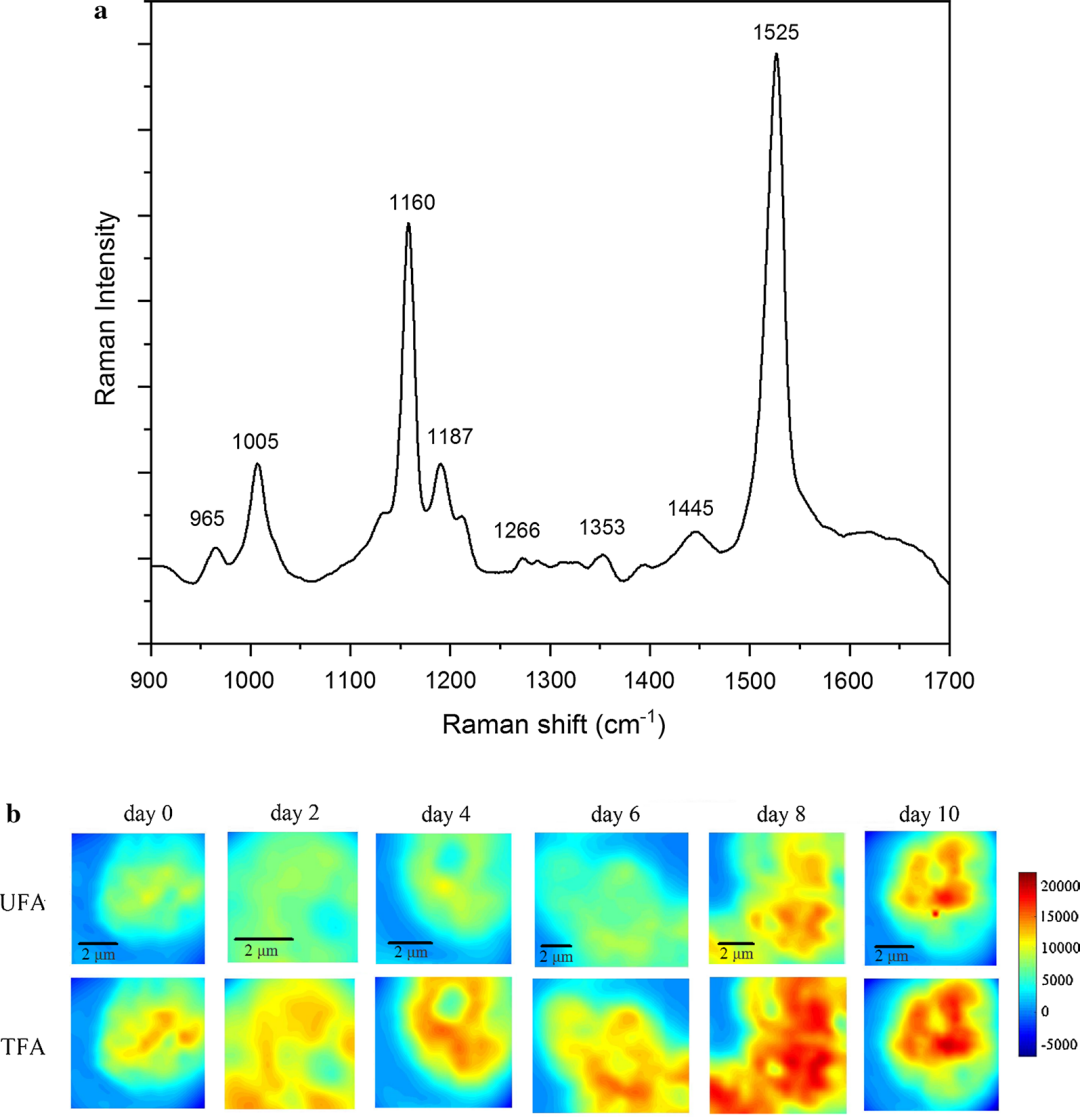
**a** Raman spectrum after baseline correction. **b** The pseudo-color maps of fatty acids distribution. (Distribution of UFA was obtained by Raman intensity at 1266 cm^−1^ and distribution of TFA was obtained by Raman intensity at 1445 cm^−1^.)

The effective band of 900 cm^−1^ to 1700 cm^−1^ was processed with the methods of cosmic rays’ removal, Savitzky–Golay smoothing and baseline correction [27–29]. As shown in Fig. 3a, the Raman spectroscopy of *S. obliquus* had a few peaks at 965, 1005, 1160, 1187, 1266, 1353, 1445, and 1525 cm^−1^, which, respectively, correspond to the metabolic components in *S. obliquus* [30–35]. Studies have shown that the corresponding H–C= bending plane at 1266 cm^−1^ mainly represented the fatty chain unsaturation [33], and the corresponding C–H_2_ bending bond at 1445 cm^−1^ mainly represented the saturated carbon chain [12, 36]. After analyzing the Raman curve above, we further performed imaging analysis, and tried comparing the results with NR and GC–MS to verify the feasibility of the results and explore the advantages of Raman in the distribution of *S. obliquus* lipid. Interpolation method was used to map the selected characteristic peaks into pseudo-color images of lipid distribution. In general, the redder the color in the captured image, the more lipids, and vice versa. As shown in Fig. 3b, the pseudo-color map of the distribution of UFA in the cells of *S. obliquus* was plotted with a peak value of 1266 cm^−1^ and the pseudo-color map of total fatty acids (TFA) distribution was plotted with the 1445 cm^−1^ peak value after correction.

It is observed from the distribution of unsaturated fatty acids in *S. obliquus* cells that the UFA are discontinuously distributed, and most of them are distributed in the two regions of the cells. The red area in the cell indicates the accumulation of UFA. The darker the color, the higher the UFA content. Comparing the NR and TEM results with the Raman results, the oil droplets were located near the cell membrane in the cytoplasm. Over time, the red area in *S. obliquus* gradually became larger and darker. It was believed that the UFA content gradually increased, and the UFA content was highest on the 8th day. The changes of pseudo-color maps of TFA and UFA are similar. The results above show that Raman spectroscopy is effective for the detection of intracellular lipid in *S. obliquus*.

### Metabolic analysis by terahertz

#### Band assignments

Some previous studies have shown that pure categories such as proteins, lipids, carbohydrates and carotenoids can be identified by THz molecular vibrational spectroscopy techniques. The main components of *S. obliquus* are protein, lipids, carbohydrate and carotenoids and their terahertz absorption spectra are distributed in the different frequency ranges: 3.3–5.0 THz for proteins [15, 37], 2.3, 9.3, 9.4, 9.8, 11.4 THz for lipids [25, 26], 9.0, 10.5, 12.1, 13.1 16.0, 17.2 and 18.0 THz for carbohydrates [16, 38], 12.1, 14.7, 15.6 and 19.6 THz for β-carotene [39]. Terahertz has been used for the study on different types of lipids and fatty acids [25, 26]. For example, saturated fatty acids (SFA) such us palmitic acid and stearic acid have distinct peaks at 9.3 and 9.4 THz, respectively. Unsaturated fatty acids (UFA), such as oleic acid, linoleic acid and linolenic acid all have two distinct peaks at 7.4 and 9.8 THz. In general, lipids usually show broader and distinct absorption peaks. In the spectra of *S. obliquus* cells, the peak of 9.3 THz corresponded to the C=O and –COO– vibration [26]. This peak mainly represented the lipids. The broad band of 3.3–5 THz corresponded to the population of overlapping discrete vibrational modes from the random amino acid sequences. It is similar to other globular proteins, such us myoglobin, hemoglobin, and lysozyme, which showed that microalgae proteins lack of highly repetitive segments in their amino acid sequences [15, 37]. The peaks of 15.6–18.0 THz mainly corresponded to the C–C–O, C–O–C, C–C–C covalent skeletal deformation [16, 38, 39]. These peaks are fused due to changes in the corresponding substance content, resulting in a significant absorption peak in the spectra of *S. obliquus*.

### Relating changes in terahertz band under nitrogen stress

**Fig. 4:**
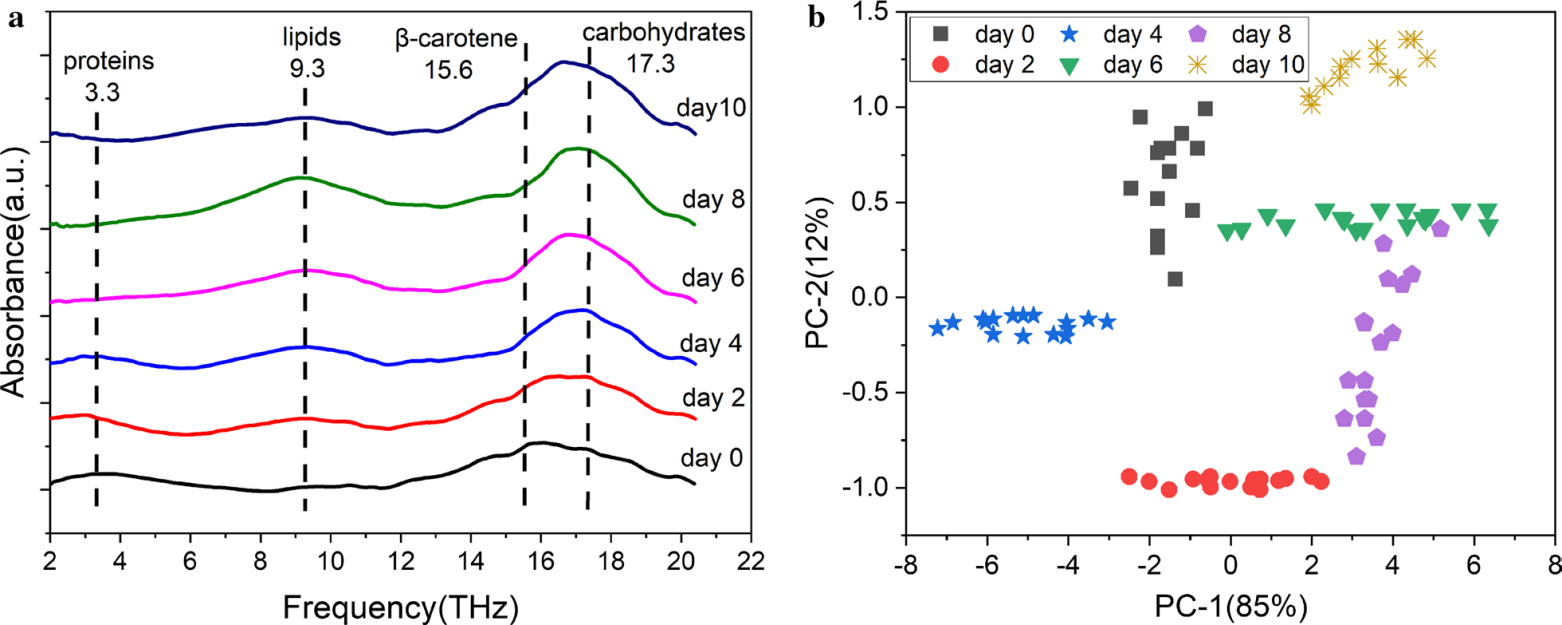
**a** Terahertz spectrum after baseline correction. **b** Score plot of PC-1 and PC-2

After confirming the corresponding absorption peak of main components in microalgae, analysis of several main absorption peaks in the *S. obliquus* spectra can reflect the changes of the main components, such as proteins, carbohydrate, lipids, carotenoids in the *S. obliquus*. Changes in the terahertz band and its corresponding components during microalgal metabolism were analyzed under nitrogen stress. During the cultivation under nitrogen stress, the protein content of the microalgae cells continues to decrease, the lipid content increased first and then decreased with its maximum on the 8th day. The carbohydrates first increased and then stabilized, and the β-carotene content of the *S. obliquus* cells continued to increase. As shown in Fig. 4a, the absorption band of 3.3–5 THz was red-shifted, which may be due to the changes in protein types of the cells. A study of microalgae showed that nitrogen starvation triggers the accumulation of multiple proteins associated with oxidative phosphorylation [4], such as NADH dehydrogenase, ATP synthase and cytochrome *c* oxidase. In general, this absorption band strength is gradually reduced because of the increase in nitrogen stress time. Absorption peaks of β-carotene and carbohydrates are difficult to distinguish at the band 15–18 THz. But from the red and blue shift of the absorption peak, the carbohydrate and β-carotene response and cumulative speed can be found. The carbohydrates rapidly accumulate, resulting in a blue shift, such as on the fourth day. The β-carotene accumulates rapidly, resulting in a red shift, such as on the tenth day. Nitrogen deficiency induces β-carotene accumulation, but the response speed is significantly slower than that of carbohydrates.

### PCA and PLS modeling based on THz spectroscopy

Spectra in the range of 2–20 THz for each day were further analyzed by PCA algorithm to distinguish these ingredients. THz spectra of the cells in different cultivation days showed obvious clustering in PC-1 and PC-2 directions (Fig. 4b). PC1 and PC2 explained in total 97% of the variation in the spectra (85% by PC1 and 12% by PC2). The aggregation of algae samples is mainly affected by protein, carbohydrate, lipid and pigment content. Among them, the decrease of protein content and the increase of lipid content have the greatest influence. Combining the results shown in Fig. 2a, b, the absorbance value of lipids changes most obviously at 0–10 days, which is consistent with the clustering effect of samples analyzed by PCA at 0–10 days. PCA analysis shows that the THz spectra of *S. obliquus* can be effectively distinguished for different growth days under nitrogen stress, and it is feasible to analyze the changes of microalgae metabolites.

Terahertz data were divided into modeling set and predicting set with a ratio of 2:1. Three different modeling schemes were used: full band modeling, lipids characteristic band modeling (2.3, 4.6, 5.3, 6.0, 9.3, 9.4, 9.8, 11.4 THz), and other characteristic band modeling (3.3, 9.0, 10.5, 12.1, 13.1, 14.7, 15.6, 16.0, 17.2, 18.0, 19.6 THz). PLS algorithm is used to model the data of modeling set to discriminate different lipid content of *S. obliquus* under nitrogen stress. When building PLS prediction model, the accuracy of prediction model is determined by comparing correlation coefficient and root mean square error of calibration set and prediction set. It can be seen from Table 2 that the lipid characteristic band modeling and full band modeling are better than other band modeling. The correlation coefficient (*r*) of the lipid characteristic band modeling is close to that of the full band modeling, but considering RMSE and RPD, the lipid characteristic band modeling is the best. These results indicated that the model constructed by terahertz spectrum of lipid characteristic band can effectively distinguish *Scenedesmus obliquus* with different lipid content under nitrogen stress, and the accuracy of the model was relatively high. On this basis, we combined terahertz bands to model and obtained better results, which proves that this prediction method was effective.

**Table 2:** Performance of PLS models for prediction of lipid content in *S. obliquus*

Discrimination model	Calibration		Validation		Prediction		
	*r*_cal_	RMSEC	*r*_val_	RMSECV	*r*_pre_	RMSEP	RPD
Full band	0.990	0.226	0.975	0.230	0.986	0.232	4.0
Lipid characteristic band	0.988	0.125	0.978	0.150	0.991	0.132	4.7
Other characteristic bands	0.945	0.492	0.919	0.510	0.934	0.523	1.8

### Quantitative analysis of lipid by lipid characteristic peak area

In the quantitative analysis of the spectrum, two methods can be used: the peak intensity of spectrum (the peak height in the spectrum) and the integration of the characteristic peak area. Next, we will carry out the quantitative analysis of the lipid of *S. obliquus* based on the characteristic peak area. In the terahertz spectrum, there is a lipid characteristic peak that changes obviously over time at 9.3 THz. In the Raman spectrum, the lipid characteristic peak of 1445 cm^−1^ is considered to be used for semi-quantitative monitoring of the lipid content of microalgae [12, 28, 36].

**Fig. 5:**
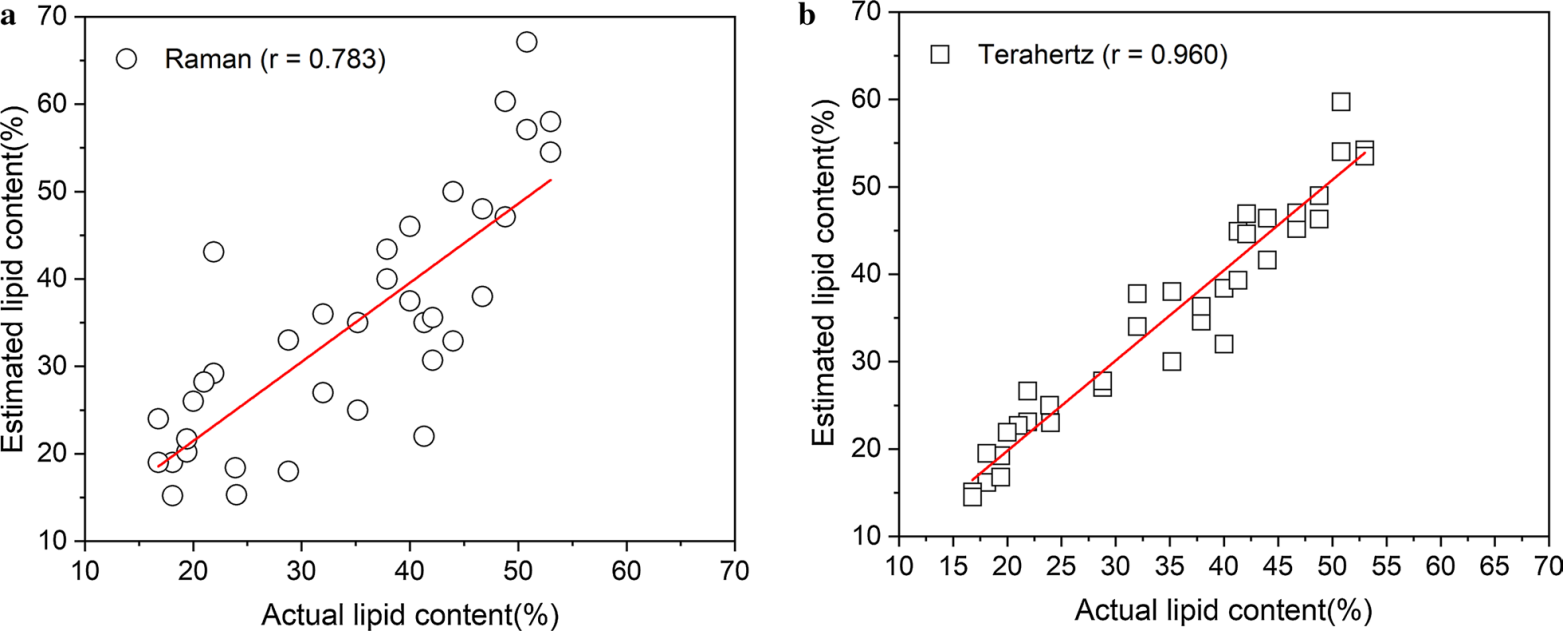
Correlation between estimated lipid content using **a** 1445 cm^−1^, **b** 9.0 THz characteristic peak area and measured lipid content obtained by gravimetric method

Figure 5 shows a comparison between estimated and actual lipid content for the same samples. The estimated lipid contents were obtained from characteristic peak areas at 9.3 THz and 1445 cm^−1^. The estimated lipid content using the characteristic peak of 9.3 THz ranged from 17.3 to 52.6% (standard error ≤ 6.3%), the estimated lipid content using the characteristic peak of 1445 cm^−1^ ranged from 18.0 to 57.2% (standard error ≤ 11.0%). The terahertz model had *r* of 0.960 and the Raman model had *r* of 0.783. For the prediction of lipid content in microalgae based on lipid characteristic peaks, the accuracy of the Raman model in this paper is similar to the results of other studies [12, 28], and the prediction accuracy of the terahertz model based on lipid characteristic peaks is higher than that of the Raman model based on lipid characteristic peaks.

## Discussion

The gravimetric method relies on solvent-based lipid extraction, which is usually time-consuming, insensitive to small differences in lipid content, and requires large amounts of cellular material and toxic solvents. In addition, since traditional gravimetric methods involve multiple steps, some neutral lipids may be lost. NR can directly observe lipid accumulation, but there are some limitations, such as spectral interference with chlorophyll autofluorescence, non-specific fluorescence and inconsistent cell absorption. TEM can clearly analyze the structure of cells and organs from a micro-perspective, but the cost is too high, which is suitable as a supplementary method. GC–MS can get the specific composition of lipids, but the sample pretreatment method is destructive, time-consuming, and not environmentally friendly and does not allow for real-time lipid content monitoring.

Terahertz spectroscopy and confocal Raman spectroscopy are both fast and non-destructive methods for microalgae lipid monitoring. When monitoring lipid changes in the cells of *S. obliquus*, the advantage of Raman spectroscopy is that it can visualize the lipids in microalgae, which helps to study the lipid accumulation process in living microalgae. The accuracy of the quantitative model of microalgae lipids by terahertz spectroscopy is better than that of Raman spectroscopy, which provides a new detection technology for real-time monitoring of microalgae lipids. Confocal Raman microscopy technology is suitable for small samples that require high-frequency screening, such as monitoring the results of genetic engineering experiments. Raman spectroscopy can measure lipid content at single cell or even single organelle resolution, revealing the heterogeneity of lipid content between organelles or between cells. When monitoring lipid changes in the cells of Scenedesmus obliquus, Raman spectroscopy can realize the visualization of lipids in microalgae, but the strong Raman signal of the fluorescent pigment overlaps with the lipid peak and affects its accuracy [12, 28]. Terahertz spectroscopy has a high signal-to-noise ratio [40]. It is a new type of spectroscopic analysis method for monitoring microalgae lipids, which is more suitable for industrial production or general laboratories that require intermediate- or low-frequency screening. When quantifying microalgae lipids, terahertz spectroscopy technology is far less restrictive than Raman spectroscopy technology, and it is easier to accurately quantify microalgae lipids, which can provide technical support for the industrial production of oil-producing microalgae under environmental control.

## Conclusions

In this paper, terahertz spectroscopy is proposed to monitor the lipid content of *S. obliquus*, PCA is used to distinguish microalgae with different stress days, and PLS is used to predict the lipid content of microalgae. The model based on the lipid characteristic band has the best performance, with a correlation coefficient of 0.991. According to the characteristic peak area at 9.3 THz, a lipid content prediction model of *S. obliquus* was established, and the correlation coefficient of the model reached 0.960. Both the intensity and area of the characteristic peak can effectively predict the lipid content of oblique algae, which proves the effectiveness of terahertz spectroscopy to monitor changes in lipid content of microalgae under nitrogen stress. Compared with Raman spectroscopy, terahertz spectroscopy is more suitable for industrial production or general laboratories that require intermediate result with low-frequency screening. When quantifying microalgae lipids, the constraint of terahertz spectroscopy technology is far less than that of Raman spectroscopy technology, and it is easier to accurately quantify microalgae lipids. In addition, it is still in the early stage for the research of microalgae using terahertz spectroscopy technology, and there is still a lot of potential for us to explore.

## Material and methods

### Culture conditions

*Scenedesmus obliquus*, FACHB-12, was purchased in Freshwater Algae Culture Collection at the Institute of Hydrobiology, FACHB-collection. *S. obliquus* was cultivated in an ultra-clean platform, in which the temperature was set at about 25 ℃, the light intensity was 2500 lx, and the light and illumination dark period was 12 h:12 h. The normal BG11 basal medium was configured according to the standards provided by the Institute of Hydrobiology.

*Scenedesmus obliquus* was expanded in BG11 medium for about 2 months and cultured to a stable growth stage, and microalgae were harvested for experiments. The algal fluid was centrifuged by a centrifuge, and the supernatant was decanted so as to reserve the bottom algal puree. And then the algal puree was washed twice with ultrapure water. Finally, the algal puree which has removed solute was harvested. 1.5 g/l NaNO_3_ in normal BG11 medium was reduced to 0.5 g/l to prepare the nitrogen-deficient medium. The harvested algal puree was cultivated in media with the 2000 ml of the algal fluid volume. The algal fluid was placed in the ultra-clean platform and the cultivation conditions were consistent with the original cultivation conditions. Before each experiment, the microalgae cell concentration was estimated by the cell count plate method to ensure that the microalgae were in stable growth throughout the experimental period.

### Gravimetric method

1. Algae powder weighing: a clean centrifugal tube was weighed (*W*_0_) and a clean Petri dish was dried to constant weight (*W*_1_). 50 ml of algae solution was centrifuged at the speed of 9000 r/min for 15 min. The liquid supernatant was removed and then the tube was dried in a 50 ℃ oven to constant weight (*W*_2_).
2. Chlorophyll destruction: the dried algal flour adding a 3 ml mixture of 5% KOH and 30% methanol was placed in a 50 ℃ water bath for 15 min. After that it was still standing for 4 h and then centrifuged at 7000 r/min for 5 min.
3. Crude lipid extraction: 2 ml water, 4 ml methanol, and 4 ml chloroform were added into the centrifuged tube and mixed for 5 min.
4. Weighing: transfer all chloroform layer to the clean Petri dish in step (1), place the dish in a fume hood at 25 ℃ to completely volatilize the chloroform, and weigh the dish again (*W*_3_).
5. Lipid content calculation: $\mathrm{lipid content }\left(\mathrm{\%}\right)=\frac{{\mathrm{w}}_{3}{-\mathrm{w}}_{1}}{{\mathrm{w}}_{2}-{\mathrm{w}}_{0}}.$

### Determination of lipid composition

1. Algal slurry harvest: 500 ml of the uniform algae of *S. obliquus* was taken after collecting the spectrum, and then was placed in centrifuge tubes separately centrifuging at a speed of 9000 r/min for 15 min. The temperature of the centrifuge was set to 4 ℃ simultaneously. After centrifugation, the supernatant was discarded, and then ultrapure water was added to the remaining algal slurry to vibrate into a uniform algae solution. The algae were then centrifuged at the same parameters, and harvested after repeating the ultrapure cleaning process. All algae harvested in the centrifuge tubes were broken by ultrasonic vibration in a 50-ml round-bottom flask for 15 min.
2. Fatty acid extraction: methanol, chloroform, and hydrochloric acid were mixed in a ratio of 10:1:1 to form a reaction mixture. 10 ml of the reaction mixture was added to the algal mud after ultrasonic wave breaking. Then the round-bottom flask containing the reactants was refluxed in a water bath at 90 ℃ for 4 h. The fatty acid was extracted by adding 3 ml of *n*-hexane, and the extraction was repeated for 3 times. The extract liquor was later transferred to a 10-ml centrifuge tube and then concentrated to 1 ml by nitrogen blowing method.
3. Chromatography–mass spectrometry conditions: using an automatic detection program, temperatures of the injection port and detector were set to 250 ℃ and 300 ℃, respectively, the split ratio was 50:1, and the temperature of the oven was controlled in following profile. It was gradually heated from 60 to 150 ℃ in a speed of 30 ℃ per minute, then it would gradually increase to 240 ℃ at the rate of 13 ℃ per minute and last for 30 min, and eventually the oven would heat up to 265 ℃ for 5 min.
4. Result analysis: the collected data were analyzed using the peak area method. And the components were determined by comparing the peaks with NIST mass spectrometry database.

### Nile red staining method

After the *S. obliquus* cell walls were broken, 0.5 ml of DMSO solution was added to 4 ml of the algae solution and mixed for 10 min. Then 40 μl of NR mother liquor (0.1 g/l) was added and stained for 30 min. Finally, the sample was observed in the dark under the fluorescence microscope.

### Raman spectroscopy collection and processing

In order to ensure the position of the microalgae cells unchanged when collecting data, the cells are fixed with agar. During the procedure, 3 g agar powder was dissolved in 100 ml of ultrapure water and then before cooling to 45 ℃ the agar liquid was boiling completely for 3 min. After mixing 3 ml agar solution with 1 ml algae solution evenly, the sections were placed on the glass slides for analyzing confocal Raman micro-spectroscopy. Firstly, microalgae cells were found by using the microscope section of the Renishaw laser confocal micro-Raman spectrometer (Renishaw PLC, United Kingdom/InVia–Reflex 532/XYZ) and were selected by the wire software for acquiring the mapping plots. The prepared sample was fixed on the objective table, the laser beam would emit with a 532 nm argon ion (Ar+) laser (power: 20 mW) and focus through a 50× objective onto the surface of the sample 16. Following were the details of the setting during the procedures where the exposure time was set to 1 s, the laser light intensity was 1%, the scanning range was 600 to 1800 (Raman Shift/cm^−1^), the scanning step length was 0.7 μm, and the cumulative number of times was one. The entire experiment was conducted at a constant temperature (about 25 ℃). Using baseline correction, Savitzky–Golay smoothing and data normalization to preprocess Raman data. Different spectral data processing uses different data normalization formulas. When calculating the Raman intensity, we sum the intensity of the marker bands in all pixels and divide by the number of pixels.

### Terahertz spectroscopy collection and processing

The cell suspension was harvested by centrifugation for 5 min at 9000 r/min at ambient temperature, and about 200 mg of algal mud was obtained. The pellets were washed twice with distilled water and the centrifugation was repeated. Then the algae solution concentrated to 1 ml was added to a smooth polyethylene mold with a diameter of 20 mm and dried for 6 h at 40 °C using a dryer for lipid characterization and biomass composition determination. At this time, the sample was prepared in the form of film with a thickness of 20 ± 5 μm. Terahertz spectra were acquired with a resolution of 0.06 THz, and each measurement is an average over 128 sample scans and 128 background scans, in the range between 2 and 20 THz using a Fourier Transform spectrometer (Vectex80v, Bruker, Germany). The light source of the far-infrared module is a water-cooled mercury lamp, and the detector is a DLaTGS/polyethylene detector. In order to eliminate the absorption contribution from atmospheric water vapor, the entire light path of instrument was a Nidec rotary vacuum pump. The experiment was performed every 2 days, six samples were measured every day and each sample was measured 5 times repeatedly. The same experiment was replicated three times, a total of 108 sets of data were obtained.

Baseline correction and Savitzky–Golay smoothing are used to preprocess the THz data. The preprocessed Raman and THz data are imported into Unscrambler v10.1. Then principal component analysis (PCA) and partial least squares (PLS) are used to classify and predict the data. PCA is an unsupervised learning method and a commonly used data processing method in multivariate statistical analysis. This method can more easily grasp the main contradictions and reduce the complexity of the data by reducing the number of dimensions [10]. The variance of the principal component is greater, and the model reflects more information. The purpose of this method is to simplify the mathematical model and improve the efficiency of data analysis. PLS is a supervised learning method and a multiple linear regression method based on input and predicted values. The correlation coefficient of the predicted value is used to find the best solution between the data and the model. Multiple regression analysis improves the accuracy of model prediction [11]. The prediction performance was evaluated by the correlation coefficient (*r*) and root mean square error of calibration (RMSEC), validation (RMSECV) or prediction (RMSEP) and residual predictive deviation (RPD). RPD value was defined as the ratio of the standard deviation of the reference data to the root mean square error (RMSE) of the calibration set during cross-validation, and it was used to evaluate how well the calibration model could predict compositional data.

## Data Availability

Not applicable.

## Abbreviations

*S. obliquus*: *Scenedesmus obliquus*
GC–MS: Gas chromatography–mass spectrometry
NR: Nile red
THz: Terahertz
IR: Infrared
TEM: Transmission electron microscopy
TFA: Total fatty acids
SFA: Saturated fatty acids
UFA: Unsaturated fatty acids
PCA: Principal component analysis
PLS: Partial least squares
RMSE: Root mean square error
